# Cryptotanshinone ameliorates CUS-induced depressive-like behaviors in mice

**DOI:** 10.1515/tnsci-2020-0198

**Published:** 2021-11-30

**Authors:** Kaixin Wang, Qingling Zhai, Sanwang Wang, Qiongyu Li, Jing Liu, Fantao Meng, Wentao Wang, Jinjie Zhang, Dan Wang, Di Zhao, Cuilan Liu, Juanjuan Dai, Chen Li, Minghu Cui, Jinbo Chen

**Affiliations:** Department of Neurology, Binzhou Medical University Hospital, No. 661 Huanghe 2nd Road, Binzhou, Shandong, 256603, China; Medical Research Center, Binzhou Medical University Hospital, Binzhou, Shandong, China; Institute for Metabolic & Neuropsychiatric Disorders, Binzhou Medical University Hospital, Binzhou, Shandong, China; Department of Internal Medicine, Jinan Hospital, Jinan, Shandong, China; Department of Psychology, Binzhou Medical University Hospital, No. 661 Huanghe 2nd Road, Binzhou, Shandong, 256603, China; Department of Gastroenterology, Binzhou Medical University Hospital, Binzhou, Shandong, China

**Keywords:** cryptotanshinone, depression, neuroinflammation, microglial polarization, neurogenesis, BDNF

## Abstract

**Objectives:**

Cryptotanshinone (CPT), a natural quinoid diterpene, isolated from *Salvia miltiorrhiza*, has shown various pharmacological properties. However, its effect on chronic unpredictable stress (CUS)-induced depression phenotypes and the underlying mechanism remain unclear. Therefore, the aim of this study was to investigate whether CPT could exert an antidepressant effect.

**Methods:**

We investigated the effects of CPT in a CUS-induced depression model and explored whether these effects were related to the anti-inflammatory and neurogenesis promoting properties by investigating the expression levels of various signaling molecules at the mRNA and protein levels.

**Results:**

Administration of CPT improved depression-like behaviors in CUS-induced mice. CPT administration increased the levels of doublecortin-positive cells and reversed the decrease in the expression levels of brain-derived neurotrophic factor (BDNF)/tyrosine kinase receptor B (TrkB) signaling transduction, as well as the downstream functional proteins, phosphorylated extracellular regulated protein kinases (p-ERK), and cyclic adenosine monophosphate (cAMP)-response element-binding protein levels (p-CREB) in hippocampus. CPT treatment also inhibited the activation of microglia and suppressed M1 microglial polarization, while promoting M2 microglial polarization by monitoring the expression levels of arginase 1 (Arg-1) and inducible nitric oxide synthase (iNOS), and further inhibited the expression of proinflammatory cytokines, including interleukin (IL)-1, IL-6, and tumor necrosis factor-α (TNF-α), and increased the expression of the anti-inflammatory cytokine IL-10 by regulating nuclear factor-κB (NF-κB) activation.

**Conclusions:**

CPT relieves the depressive-like state in CUS-induced mice by enhancing neurogenesis and inhibiting inflammation through the BDNF/TrkB and NF-κB pathways and could therefore serve as a promising candidate for the treatment of depression.

## 1 Introduction

Depression, one of the most prevalent psychiatric disorders, is a significant public health problem globally and places a substantial burden on socioeconomic development [1]. In recent years, the prevalence of depression has been increasing annually [2]. Traditional antidepressant treatments include selective serotonin reuptake inhibitors, norepinephrine reuptake inhibitors, tricyclic antidepressants, and monoamine oxidase inhibitors, which generally require a treatment cycle of several weeks to several months, and nearly 50% of patients have very poor or no response to these drugs [3]. Therefore, it is imperative to identify new antidepressant drugs to achieve more effective treatment goals [1,4].

Studies have shown that neurogenesis and neuroinflammation play important roles in the pathogenesis of depression [5,6,7]. Chronic, uncontrollable, and unpredictable stress can trigger discomfort in humans and animals, leading to the occurrence and development of depression [8,9]. In the brain, neurogenesis is a key repaired response to stress [10]. Neurogenesis is mainly promoted by neural progenitor cells located in the granular zone of the dentate gyrus (DG) to produce new neurons. After these new neurons are generated, they proliferate, migrate, and differentiate and are subsequently integrated into the existing neuronal circuits to form synaptic connections [11]. Brain-derived neurotrophic factor (BDNF) is widely distributed in the brain and plays an important role in the survival, development, and growth of central neurons [12] and is involved in the regulation of neurogenesis [13]. Decreased BDNF levels and abnormal neurogenesis are associated with depressive symptoms and behaviors [14,15,16].

Neuroinflammation is also a key cause of the onset, exacerbation, and recurrence of depression [17]. A prior study found that a significant increase in cytokines could be detected in the serum, cerebrospinal fluid, and hippocampus of patients with depression [18]. Microglias are immune cells that are present in the central nervous system, where they act as “scavengers” to maintain homeostasis. Microglia can be rapidly activated when the microenvironment changes in response to pathogen invasion and danger signals [19]. Activation of microglia produces two polarized phenotypes with very different functions: classically activated M1 (proinflammatory) and alternatively activated M2 (anti-inflammatory) macrophages [20]. Adjusting the balance between these two polarization states can prevent or delay the occurrence and development of depression [21]. Therefore, regulating activated microglia may be a potential therapeutic target for the treatment of depression.

Cryptotanshinone (CPT) is a natural quinoid diterpene isolated from the dried roots and rhizomes of *Salvia miltiorrhiza* bunge, which has been shown to exhibit various pharmacological effects, including anticancer, anti-inflammatory, antibacterial, antioxidant, and neuroprotective effects [22,23,24]. Furthermore, it has been demonstrated to have beneficial therapeutic effects against various diseases, but its effect on chronic unpredictable stress (CUS)-induced depression phenotype and its mechanisms of action are still unclear. In this study, CUS was used to establish a mouse depression model to study the antidepressant effects of CPT. To better understand its mechanism of action, we studied the effects of CPT treatment on CUS-induced neuroinflammation and neurogenesis and detected the expression of signaling pathway-relevant proteins in the hippocampus, which could provide a theoretical basis for the development of new antidepressant drugs.

## 2 Material and methods

### 2.1 Animals

Male C57BL/6 mice (8 weeks, 20–25 g) were purchased from Jinan Pengyue Experimental Animal Breeding Co., Ltd. (China; License No. SYXK (Lu) 20190003). The mice were bred under controlled temperature (22 ± 2°C) and relative humidity (55 ± 5%), with a 12 h light–dark cycle, and were allowed access to food and water *ad libitum*.


**Ethical approval:** The research related to animals’ use has been complied with all the relevant national regulations and institutional policies for the care and use of animals. All animal experimental protocols were approved by the Animal Experimental Ethics Committee of Binzhou Medical University Hospital (Binzhou, China).

### 2.2 Experimental design for drug treatment

**Figure 1:**
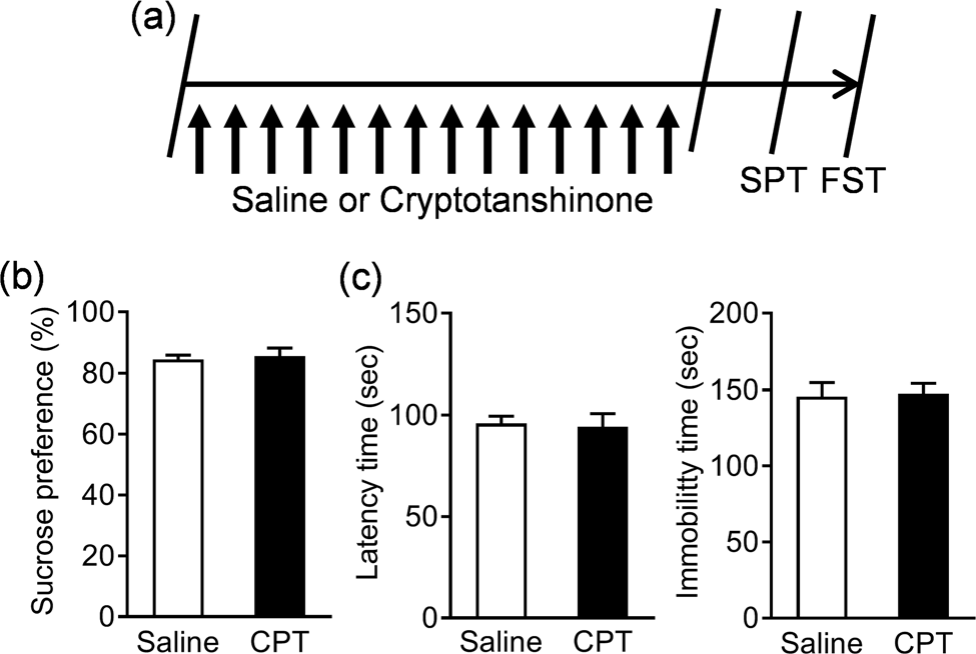
Effect of CPT on depressive behavior in basal conditions: (a) schematic of the experiment design, (b) SPT, and (c) FST. *n* = 8 per group.

**Figure 2:**
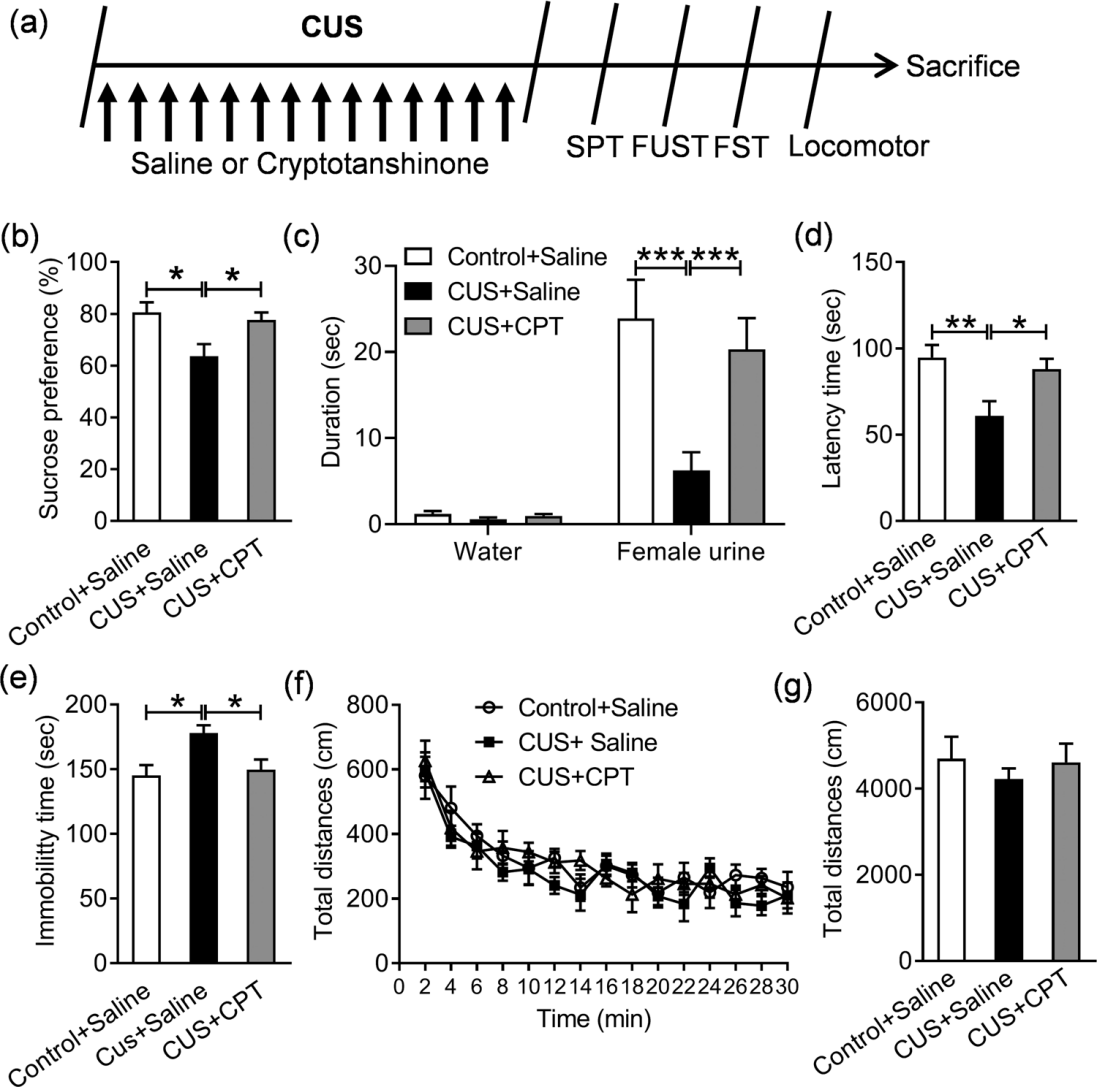
Effect of CPT on CUS-induced depressive behavior in mice: (a) schematic of the experiment design, (b) SPT, (c) FUST, (d and e) FST, and (f and g) locomotor activity test. *n* = 8 per group. *p < 0.05, **p < 0.01, and ***p < 0.001.

The experimental design and drug treatment schedule are shown in Figures 1a and 2a, wherein the effect of CPT on depressive behaviors in basal and stress conditions was evaluated independently. First, the mice were randomly divided into two groups, with eight animals per group: saline and CPT (20 mg/kg). CPT (purity ≥98%, Sigma-Aldrich, USA) was dissolved in sterile normal saline and administered by gastric gavage at a dose of 20 mg/kg body weight for 14 days. Then, the sucrose preference test (SPT) and forced swim test (FST) were conducted. Second, the mice were randomly divided into three groups, with eight animals per group: control + saline, CUS + saline, and CUS + CPT (20 mg/kg). CPT was administered by gastric gavage at a dose of 20 mg/kg body weight. The CUS + saline and CUS + CPT groups were subjected to CUS for 14 consecutive days. Simultaneously, an equal volume of saline or CTP was injected intragastrically before daily stress. CUS was performed in accordance with the methods reported in a previous study [25]. The stress included restraint for 1 h, shaking and crowding for 1 h, clip tail for 20 min, 24 h of light, swimming in cold water at 8°C for 5 min, wet cage housing for 4 h, and electric shock for 10 min. Mice were randomly assigned to one of the above stresses every day to ensure the unpredictability of the experiment. At the end of the CUS procedure, SPT, female urine sniffing test (FUST), FST, and locomotor activity were carried out in sequence. In the end, the mice were sacrificed by decapitation, and their brains were immediately collected on an ice plate. After rinsing with normal saline, the left and right hippocampus of the mice were quickly removed, placed in Eppendorf tubes, flash-frozen in liquid nitrogen, and stored at −80°C.

### 2.3 SPT

Anhedonia is one of the core symptoms of depression in humans. In this experiment, the sucrose preference experiment was primarily used to evaluate the degree of anhedonia in mice. During the entire CUS procedure, control and experimental mice were given two bottles of pure water for adaptation to avoid side preference. Water was restricted for 4 h before the experiment, and the mice were kept in a single cage with access to two bottles: one with pure water and the other with 1% (w/v) sucrose solution. Fluid consumption was recorded by weight. Finally, the sucrose preference value was calculated using the following formula:

\text{Sucrose}\hspace{.25em}\text{preference}\hspace{.25em}\text{value}=\frac{\text{Sucrose}\hspace{.25em}\text{consumption}}{\text{Sucrose}\hspace{.25em}\text{consumption}+\text{water}\hspace{.25em}\text{consumption}}]

### 2.4 FST

Mice were kept in glass cylindrical containers (25 cm height × 10 cm diameter) filled with tap water to a height of 15 cm from the bottom (maintained at 24 ± 1°C). Each mouse was recorded for 6 min, and the time to first immobility during the first 2 min and immobility in the last 4 min were recorded. Immobility is defined as the state in which the mouse floated on the surface of the water, showing only slight movements necessary to maintain breathing and floating [26].

### 2.5 FUST

FUST is an experiment that measures reward-seeking behavior in rodents based on their interest in the urine odor of the opposite sex. The experimental procedure was performed as previously described [27]. Briefly, urine from estrus female mice was collected and stored on ice before use. Prior to testing, the sterile swabs were suspended in the cage for acclimatization. During the test, the swab soaked in sterile water was exposed to the cage for 3 min. 45 min later, the swab soaked in female urine was exposed to the cage for 3 min. The test was video recorded, and the total time the mice spent sniffing the cotton swabs soaked with urine or water was measured.

### 2.6 Locomotor activity

The locomotor activity was measured in a SuperFlex Fusion open field cage (40 cm × 40 cm × 30 cm, Omnitech Electronics Inc., Columbus, OH). Individual mice was allowed to acclimate to the single cage for 2–4 h prior to the test. The animals were gently placed in the center of the test apparatus (40 cm × 40 cm × 40 cm) and allowed to explore the field for 30 min. Locomotor activity of the mice was monitored and recorded with infrared motion sensors mounted on top of the cage. The total distance traveled was analyzed using Fusion software (Omnitech Electronics Inc., Columbus, OH).

### 2.7 Quantitative real-time PCR (Q-PCR)

The total RNA was extracted from the tissue using a Total RNA Kit (Omega, Guangzhou, China), according to the manufacturer’s instructions. RNA was reverse transcribed to generate cDNA using the Reverse Transcription Kit (Takara Bio, Inc.). Q-PCR was performed using SYBR Green PCR Master Mix (Vazyme Biotech, Nanjing, China). Cycling conditions were 5 min at 95°C, followed by 10 s at 95°C and 30 s at 60°C for 40 cycles; the primer sequences are listed in Table 1. The expression level of each sample was calculated according to the threshold cycle (CT), and the relative expression was calculated using the 2^−ΔΔCT^ method [28,29].

**Table 1:** Primer sequences used for Q-PCR

Gene	Forward primer (5'–3')	Reverse primer (5'–3')
β-Tubulin	AGCAACATGAATGACCTGGTG	GCTTTCCCTAACCTGCTTGG
BDNF	CCCTGGCTGACACTTTTGAG	TCCAGCAGAAAGAGCAGAGG
Exon I	CCTGCATCTGTTGGGGAGAC	GCCTTGTCCGTGGACGTTTA
Exon II	CTAGCCACCGGGGTGGTGTAA	AGGATGGTCATCACTCTTCTC
Exon IV	CAGAGCAGCTGCCTTGATGTT	GCCTTGTCCGTGGACGTTTA
Exon VI	CTGGGAGGCTTTGATGAGAC	GCCTTCATGCAACCGAAGTA
iNOS	CAAGAGTTTGACCAGAGGACC	TGGAACCACTCGTACTTGGGA
TNF-α	CCTATGTCTCAGCCTCTTCT	CCTGGTATGAGATAGCAAAT
IL-1β	GGCAACTGTTCCTGAACTCAACTG	CCATTGAGGTGGAGAGCTTTCAGC
IL-6	CCACTTCACAAGTCGGAGGCTT	CCAGCTTATCTGTTAGGAGA
IL-10	GCCAGTACAGCCGGGAAGACAATA	GCCTTGTAGACACCTTGGTCTT
Arg-1	CTTGCGAGACGTAGACCCTG	TGAGTTCCGAAGCAAGCCAA

### 2.8 Western blot (WB)

Total protein from hippocampal tissues was extracted using radio immunoprecipitation assays lysis buffer (Beyotime, China) containing the phosphatase inhibitor PhosStop (Roche, Germany) and protease inhibitor phenylmethyl sulfonylfluoride (Beyotime, Nanjing, China). Lysates were homogenized with a tissue homogenizer and incubated on ice for 30 min. After centrifugation at 12,000 ×*g* for 15 min at 4°C, the supernatants were collected. The protein concentration of each sample was determined using the bicinchoninic acid protein assay kit to allow equal loading of total protein. Equal amounts of protein samples were loaded in 8–15% sodium dodecyl sulfate-polyacrylamide gel for electrophoresis, transferred to a PVDF membrane at 100 V for 90 min and then blocked with 5% nonfat dry milk for 2 h at room temperature under shaking conditions. After three washes in TBST (Tris-Buffered Saline, 0.1 % Tween-20) buffer, primary antibodies anti-BDNF (ab108319, 1:1,000, Abcam, Cambridge, UK), anti-ERK1/2 (#9102, 1:1,000, Cell Signaling), anti-p-ERK1/2 (Thr202/Tyr204; #4370, 1:1,000, Cell Signaling), anti-TrkB (#4603, 1:1,000, Cell Signaling), anti-p-TrkB (Tyr516; #4619, 1:1,000, Cell Signaling), anti-β-actin (#4970, 1:1,000, Cell Signaling), anti-CREB (#9197, 1:1,000, Cell Signaling), anti-cAMP-response element-binding protein levels (anti-p-CREB; Ser133; #9198, 1:1,000, Cell Signaling), anti-arginase 1 (anti-Arg-1; GTX109242, 1:500, Gene Tex, USA), anti-inducible nitric oxide synthase (anti-iNOS; ab15323, 1:500, Abcam, Cambridge, UK), anti-NF-kB (ab276, 1:500, BOSTER, China), and anti-p-NF-kB p65 (3033T, 1:1,000, Cell Signaling) were added and allowed to incubate at 4°C overnight with gentle rocking. The membranes were washed with TBST and incubated with goat anti-rabbit IR Dye 680LT (#926-68021, 1:5,000, Li-COR Biosciences, Lincoln, NE, USA) or goat anti-mouse IR Dye 800CW (#926-32210, 1:5,000, Li-COR Biosciences) fluorescent secondary antibodies for 90 min at room temperature and visualized using an Odyssey Infrared Imaging System (Li-COR Biosciences).

### 2.9 Immunofluorescence (IF)

IF experiments were performed as described previously [30]. Brains were collected from mice after transcardiac perfusion with phosphate-buffered saline (PBS) and 4% paraformaldehyde, postfixed in 4% paraformaldehyde, and dehydrated in 30% sucrose at 4°C overnight. Frozen sections of brain tissues with a thickness of 40 μm were prepared from brains embedded in the optimal cutting temperature compounds. The sections were washed four times for 5 min each time in PBS and then blocked for 1 h in blocking solution (1% bovine serum albumin, 3% Triton X-100, and 0.3% donkey serum in PBS) and incubated with anti-doublecortin (DCX) primary antibody (ab18723, 1:1,000, Abcam, Cambridge, UK) and anti-Iba-1 primary antibody (ab5076, 1:1,000, Abcam, Cambridge, UK) in blocking solution overnight at 4°C. The sections were washed with PBS (four washes, 5 min each) before incubation with a mixture of Alexa Fluor^®^ 546 goat anti-rabbit IgG antibodies (1:400, Invitrogen, Carlsbad, CA, USA) or Alexa Fluor^®^ 546 rabbit anti-goat IgG antibodies (1:300, Invitrogen) for 4 h at room temperature, and then stained with 4,6-diamino-2-phenyl indole for 15 min and washed three times with PBS. The sections were mounted onto poly lysine-coated glass slides, cover-slipped using fluorescence mounting medium, and observed under a confocal fluorescent microscope and photographed. The immunofluorescent positive cells on both sides of the dentate gyrus (DG) were counted manually.

### 2.10 Statistical analyses

Statistical analyses were performed using GraphPad Prism 8.0. The Shapiro–Wilk test and *F* test were used to test the normality and equal variance assumptions. The two-tailed *t*-tests were conducted for normally distributed data; Mann–Whitney *U* test was applied to analyze the nonnormally distributed data. The statistical significance of data over the three groups was performed using one-way ANOVA followed by Sidak *post hoc* tests. For exons or locomotor activity testing data, two-way repeated-measures ANOVA followed by Tukey’s test was used. All variables are expressed as the mean ± standard error (sem). Statistical significance was set at *p* < 0.05.

## 3 Results

### 3.1 CPT attenuated CUS-induced depressive-like behaviors

The SPT is commonly used to assess anhedonia, a core symptom of depression. We first tested the effect of drug (CPT) on the depression-related behaviors under basal conditions, and the results showed that CPT treatment has no obvious regulatory activity on the sucrose preference (Mann–Whitney test, *p* = 0.279; Figure 1b). The FST was used as a measure of the degree of despair in depression. The results showed that the latency time (*t* (14) = 0.221, *p* = 0.8286) and immobility time (*t* (14) = 0.179, *p* = 0.8608) were not significantly altered in the CPT-treated mice compared to the control mice (Figure 1c).

Furthermore, as demonstrated in Figure 2b, there was a significant treatment effect on sucrose preference (*F* (2,21) = 5.377, *p* < 0.05), and a pronounced decrease in the percentage of sucrose preference in CUS-treated mice was found (*p* < 0.05) compared with control mice, and CPT injection significantly attenuated the CUS-induced decrease in sucrose preference (*p* < 0.05). FUST was used to evaluate the reward-category activity of CUS-induced mouse depression model mice. Our results indicated that there were significant effects on CPT treatment (*F*(2,21) = 6.520, *p* = 0.006), sniffing objects (*F*(1,21) = 67.530, *p* < 0.001), and treatment × sniffing objects interaction (*F*(2,21) = 7.232, *p* = 0.004), and further analysis showed that CUS obviously decreased the sniffing duration (*p* < 0.001), which was restored by CPT (*p* < 0.001), whereas no differences in water sniffing time were observed between the three groups (Figure 2c, *p* > 0.05, *p* > 0.05).

Meanwhile, as shown in Figure 2d and e, CUS exposure effectively triggered depression-like behaviors in mice, as indicated by the decreased latency time (*p* < 0.01) and increased immobility time (*p* < 0.05) compared to control mice (*F*(2,21) = 6.017, *p* < 0.01). CPT treatment increased this latency time (*p* < 0.05) and decreased the immobility time (*p* < 0.05; *F*(2,21) = 5.925, *p* < 0.01), indicating that CPT improved CUS-induced behavioral despair in the FST. Moreover, the locomotor activity was evaluated to exclude nonspecific motor activity, and the results indicated that there was no significant difference among the groups (Figure 2f and g; treatment, *F*(2,21) = 0.377, *p* > 0.05; timepoint, *F*(14,294) = 28.260, *p* < 0.001; treatment × timepoint, *F*(28, 294) = 1.086, *p* > 0.05; and total distance, *F*(2,21) = 0.377, *p* > 0.05).

### 3.2 CPT rescued impaired neurogenesis in DG of the hippocampus

**Figure 3:**
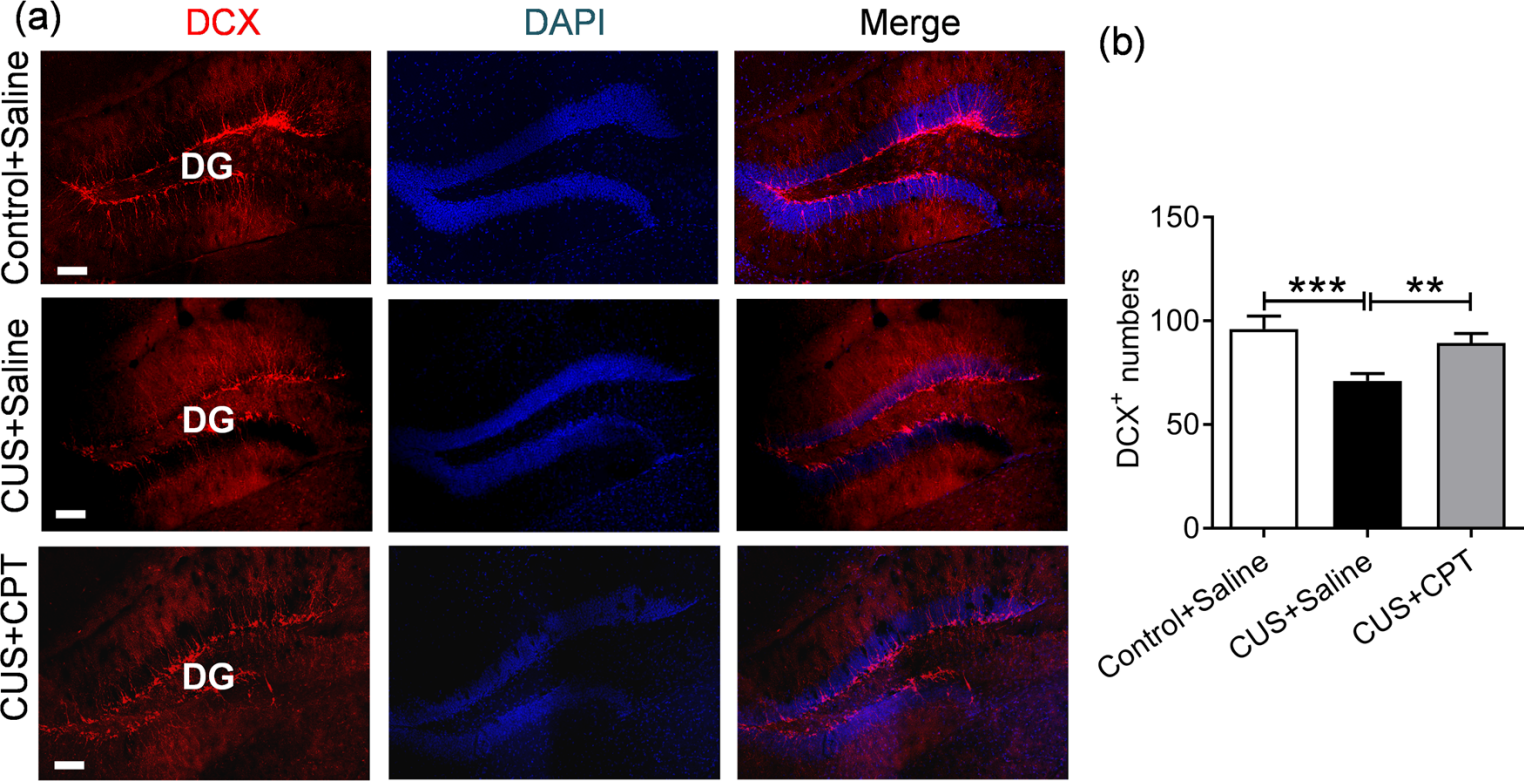
Regulation of DCX-labeled neurons in CUS- or CPT-treated mice: (a) representative immunofluorescent images and (b) quantitative statistics of DCX-positive cells, scale bar = 100 μm. *n* = 9 sections from three mice per group. **p < 0.01 and ***p < 0.001.

Impaired adult hippocampal neurogenesis has been implicated in the pathogenesis of depression. Therefore, we evaluated the ability of CPT to recover impaired neurogenesis in a CUS-induced depression model. The number of cells in DG labeled with DCX, a neurogenesis marker used to visualize neonatal and immature neurons, was significantly lower in the CUS mice than in the control mice (*p* < 0.01), whereas the number of DCX ^+^ cells was increased significantly in CPT-treated mice (*p* < 0.05). These data revealed that CUS significantly decreased the number of DCX-positive cells in DG, which could be reversed by CPT (*F*(2, 24) = 6.875, *p* < 0.01; Figure 3a and b).

### 3.3 CPT reversed the impaired BDNF/TrkB signaling transduction

**Figure 4:**
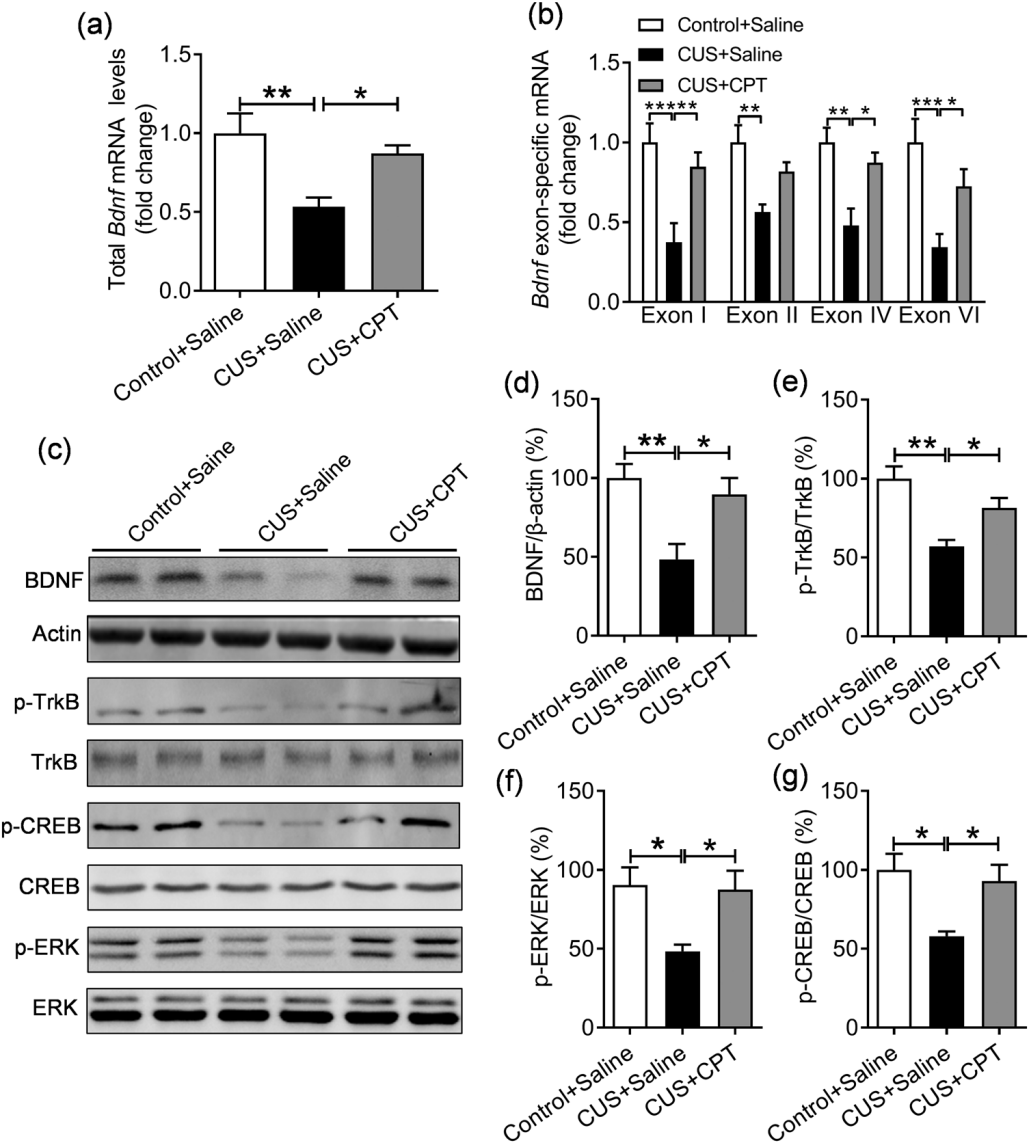
Effect of CPT on the expression of BDNF and activity of TrkB, CREB, and ERK in the hippocampus of CUS mice: (a) total *Bdnf* mRNA; (b) exon-specific *Bdnf* mRNA; (c) immunoblots showing the protein levels of BDNF, p-TrkB, p-CREB, and p-ERK; and (d–g) quantitative statistics of BDNF, p-TrkB, p-CREB, and p-ERK protein levels. *n* = 5 per group. *p < 0.05, **p < 0.01, and ***p < 0.001.

To investigate the signal transduction pathways mediating neurogenesis, further research focused on BDNF/TrkB signaling, which has been reported to be associated with the pathogenic mechanisms underlying depression. Therefore, we first measured the mRNA expression of *Bdnf* and its corresponding spliced exons using Q-PCR. CPT restored the decreased total mRNA expression of *Bdnf* in CUS-treated mice (Figure 4a, *F*(2,12) = 7.929, *p* < 0.01). Further analysis showed that the exon I (*p* < 0.001), exon II (*p* < 0.01), exon IV (*p* < 0.01), and exon VI (*p* < 0.001) mRNA levels in the CUS + saline group were decreased compared to the control + saline group, whereas the expression levels of exon I (*p* < 0.01), exon IV (*p* < 0.05), and exon VI (*p* < 0.05) mRNA levels in the CUS + CPT group were significantly increased compared with the CUS + saline group except for exon II (*p* > 0.05) (Figure 4b, treatment, *F*(2,12) = 12.580, *p* < 0.05; exons, *F*(3,36) = 1.526, *p* > 0.05; and treatment × exons, *F*(6,36) = 0.841, *p* > 0.05). WB results further verified that the CUS significantly reduced the protein levels of the hippocampal BDNF (*p* < 0.01), whereas the expression levels of hippocampal BDNF was increased after treatment with CPT (*p* < 0.05; Figure 4c and d, *F*(2,12) = 8.053, *p* < 0.01). The biological actions of BDNF are transduced through TrkB receptor [31,32]. Therefore, we next examined the levels of activation of TrkB, which was assessed by the levels of tyrosine phosphorylation, the results showed that CUS significantly reduced the phosphorylation of TrkB (*p* < 0.01), which was reversed by CPT treatment (*p* < 0.05; Figure 4c and e, *F*(2,12) = 11.960, *p* < 0.01).

To confirm the activation of BDNF/TrkB signaling transduction, we further tested the activation of ERK, an important signaling transduction target of BDNF/TrkB [31]. WB analysis showed that the expression of p-ERK in the hippocampus was significantly reduced by CUS (*p* < 0.05), whereas the level of p-ERK in the CPT treatment group increased (*p* < 0.05; Figure 4c and f, *F*(2,12) = 5.7460, *p* < 0.05). In addition, changes in p-CREB, a key transcription factor responsible for BDNF expression [33], were also detected. We found that CUS caused a significant decrease in hippocampal p-CREB (*p* < 0.05), whereas the level of p-CREB was increased in the CPT treatment group (*p* < 0.05; Figure 4c and g, *F*(2,12) = 6.9270, *p* < 0.01).

### 3.4 CPT reversed microglial activation and the release of proinflammatory factors induced by CUS

**Figure 5:**
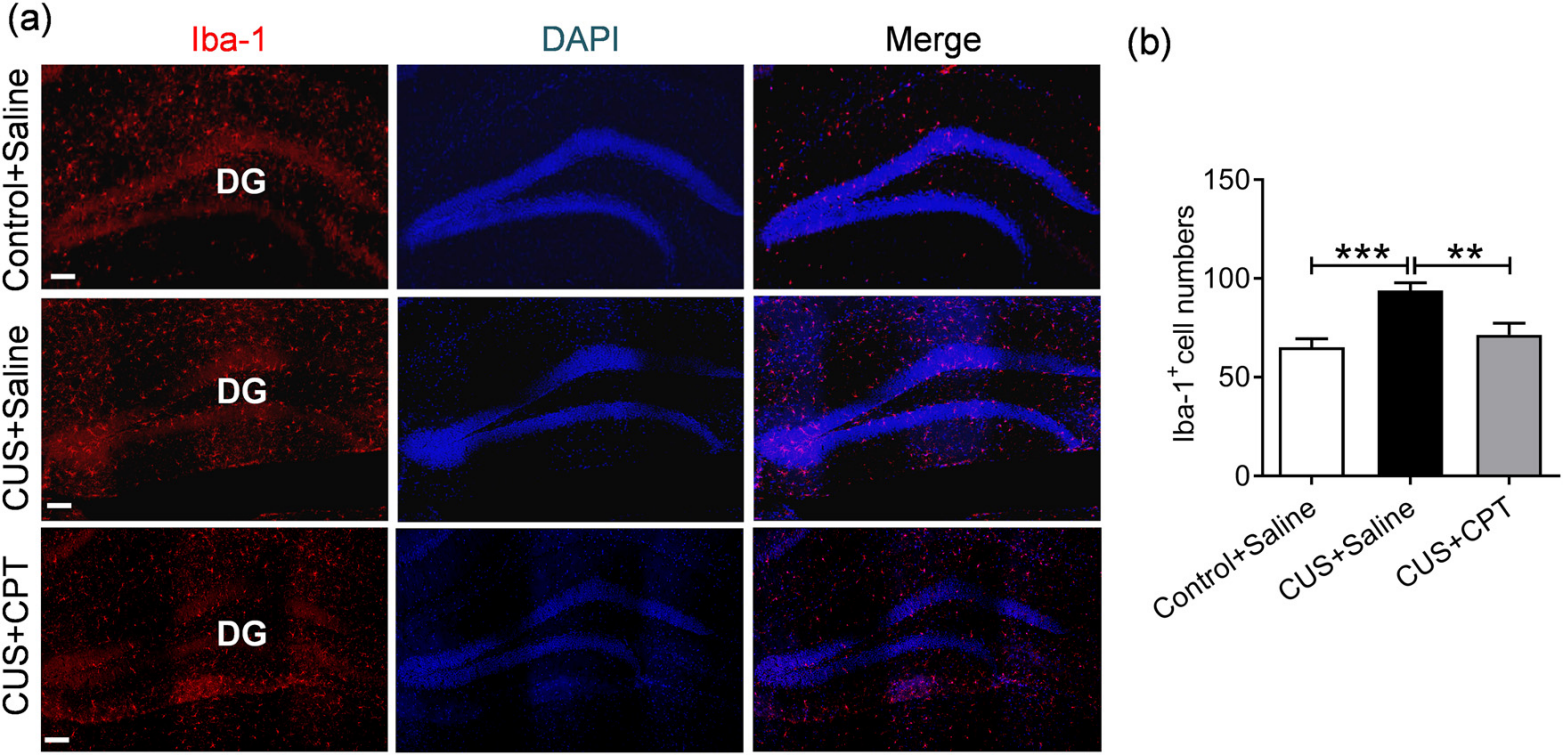
Regulation of Iba-1-labeled microglia in CUS- or CPT-treated mice: (a) representative immunofluorescent images and (b) quantitative statistics of Iba-1-labeled microglia, scale bar = 100 μm. *n* = 9 sections from three mice per group. **p < 0.01 and ***p < 0.001.

**Figure 6:**
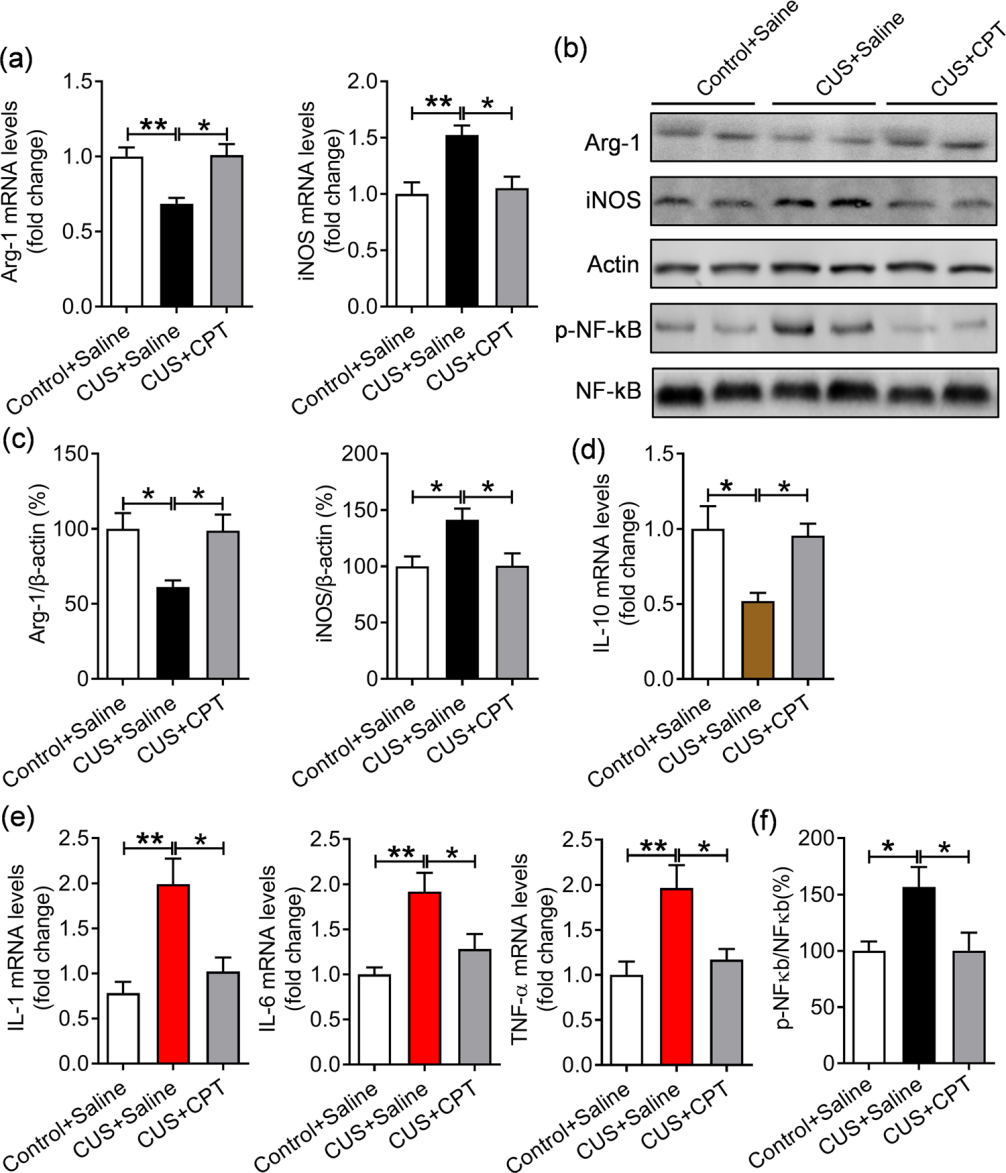
Effect of CPT on CUS-induced inflammatory factors and NF-kB activation in the hippocampus: (a) mRNA levels of Arg-1 and iNOS, (b) immunoblots showing the protein levels of Arg-1, iNOS, and p-NF-κB, (c) quantitative statistics of Arg-1 and iNOS protein levels, (d) mRNA levels of IL-10, (e) mRNA levels of IL-1, IL-6, and TNF-α, and (f) quantitative statistics of p-NF-κB protein levels. *n* = 5 per group. *p < 0.05 and **p < 0.01.

Neuroinflammation is thought to be one of the key factors involved in the etiology of depression [34]. The microglial activation and subsequent neuroinflammatory response are closely related to neuroinflammation [35]. To verify the effect of CPT on microglia, we used IF to detect the expression of Iba-1, a marker of microglial activation. We found that CUS stimulation significantly increased the number of Iba-1-positive cells (*p* < 0.001), whereas the expression level of Iba-1-positive cells was obviously decreased after CPT treatment (*p* < 0.01; Figure 5a and b, *F*(2,24) = 9.815, *p* < 0.001). Similarly, the levels of Arg-1 and iNOS, markers of microglia/macrophage polarization, were measured to further elucidate the mechanism underlying the anti-inflammatory effect of CPT. As shown in Figure 6a–c, CUS induced a significant decrease in hippocampal Arg-1 mRNA and protein expression (*p* < 0.01 and *p* < 0.05) and a significant increase in iNOS expression (*p* < 0.01 and *p* < 0.05), whereas CPT treatment group reversed the changes in Arg-1 (*p* < 0.05 and *p* < 0.05) and iNOS (*p* < 0.05 and *p* < 0.05; Arg-1: mRNA, *F*(2,12) = 9.842, *p* < 0.01; protein, *F*(2,12) = 5.970, *p* < 0.05; iNOS: mRNA, *F*(2,12) = 8.665, *p* < 0.01; protein, *F*(2,12) = 5.584, *p* < 0.05). This finding indicated that CPT increases the polarization of M2-type microglia/macrophages, decrease the prevalence of M1-type microglia/macrophages, and promoted the polarization of M1 microglia/macrophages to the M2 phenotype.

Moreover, the mRNA expression of IL-10, IL-1, IL-6, and TNF-α in the hippocampus was measured, and it was found that CUS stimulation decreased the mRNA expression of the anti-inflammatory factors Arg-1 and IL-10, whereas increasing the expression of the proinflammatory factors IL-1, IL-6, TNF-a, and iNOS, while CPT treatment restored these abnormal changes (Figure 6d and e; IL-10: *F*(2,12) = 6.571, *p* < 0.05; IL-1: *F*(2,12) = 10.18, *p* < 0.01; IL-6: *F*(2,12) = 8.756, *p* < 0.01; TNF-a: *F*(2,12) = 7.873, *p* < 0.01). Furthermore, to confirm the regulatory mechanism of CPT on the inflammatory mediators, the activity of NF-κB, a central inflammatory and proinflammatory factor, was measured, and our results showed that the levels of p-NF-κB were increased by CUS (*p* < 0.05), and this effect was attenuated by CPT (*p* < 0.05; Figure 6f, *F*(2,12) = 5.0050, *p* < 0.05).

## 4 Discussion

CUS can be applied to mice to produce an animal model of induced depression-like behavior. This model is widely used for its strong efficacy and reliable predictability. In this study, we used the CUS-induced depression models to evaluate the antidepressant activity of CPT, a natural compound, which is one of the main medicinal active ingredients of Danshen (a traditional medicinal ingredient in China). We found that CPT alleviated the CUS induced depression-related phenotypes by promoting neurogenesis and BDNF/TrkB signaling and inhibiting microglial activation and the release of proinflammatory factors in CUS mice.

The hippocampus is a key brain region for mood regulation. At the same time, studies have shown that neurogenesis in the hippocampus is one of the most unique phenomena in the mammalian brain [36]. Neurogenesis plays an important role in the pathogenesis of depression and the response to antidepressants [5,37]. DCX is only expressed in the first 3 weeks of new neurons [38]. Studies have reported that DCX-positive neurons mainly occur in the hippocampal DG, whereas DCX immunoreactivity is hardly found in CA1 and CA3, indicating that neurogenesis mainly occurs in the DG and less so in the CA1 and CA3 [36]. This is consistent with what we observed in our results of DCX IF staining, showing that CPT increased the neurogenesis in the hippocampal DG, suggesting that CPT may exert antidepressant effects by improving neurogenesis.

BDNF is highly expressed in the hippocampus and is sensitive to the stress response, which can be used as a biological marker of the pathogenesis and course of depression [39,40]. The present study showed that CPT upregulated BDNF mRNA and protein expression, as well as components of its downstream transduction pathway, implying that the antidepressant activity of CPT may be mediated by BDNF signaling. Transcription of the mouse *Bdnf* gene is controlled by nine different promoters, which results in the expression of different BDNF transcripts encoding an identical protein [41,42]. Under different stresses and stimuli, the expression of *Bdnf* is regulated by individual exons/promoters [43]. Some studies have shown that antidepressants can lead to different expressions of certain exon-specific transcripts [44,45,46]. Meanwhile, we found that exons I, II, IV, and VI were obviously decreased by CUS, which has been widely reported to act as functional exons implicated in psychiatric disorders [47], whereas CPT reversed the decreases in BDNF transcription by enhancing the expression of exons I, IV, and VI, which indicates that the complex regulation mechanisms of CUS-induced pathogenesis and CPT were involved in therapeutic activity of depression, which may be a consequence of their distinct subcellular localization, spatially restricted effects, or the regulation of different signaling pathways [48]. The BDNF/TrkB signaling pathway provides a favorable microenvironment for neuronal survival, neurodevelopment, and neurogenesis. There is increasing evidence linking impaired neurogenesis and neurotrophic factor deficiency to the occurrence and development of depression [49]. The data of this study showed that the BDNF/TrkB pathway was impaired during CUS stimulation, and CPT treatment restored the expression level of BDNF/TrkB. At the same time, we found that p-ERK and p-CREB, which are downstream signaling pathways of BDNF/TrkB, were also impaired in the hippocampus of CUS-induced mice. CPT treatment restored the decreased expression levels of p-ERK and p-CREB, indicating that the antidepressant effect of CPT seems to be mediated by the BDNF/TrkB signaling pathway.

The relationship between neuroinflammation and depression has been widely recognized [35]. Microglias are the main effectors of neuroinflammation, which are quickly activated in response to various endogenous or exogenous stimuli [50]. Our study found that CPT treatment can inhibit the activation of microglia induced by CUS, implying that microglia-associated neuroinflammation may be another target for the antidepressant effect of CPT. When responding to a variety of environments, the phenotypes and functions of activated microglia are highly plastic and diverse and are usually divided into two functionally distinct phenotypes, composed of phenotypes of “classical” activation (M1) and “alternative” activation (M2) [51]. M1-type microglias can produce proinflammatory factors, inflammatory mediators, etc., which have toxic effects on neurons, thereby promoting tissue inflammation and damage, leading to neurotrophic system dysfunction [52]. In contrast, M2-type microglia release anti-inflammatory mediators and neurotrophic factors to combat central nervous system damage caused by inflammation [53]. iNOS is a specific marker of M1-type microglia, and Arg1 is a specific marker of M2-type microglia, which is considered to be a special phenotype of beneficial microglia. Arg-1 cells regulate inflammation in the brain and promote synaptic growth, thereby exerting neuroprotective effects [54,55]. In this study, we observed that CPT decreased the expression of the M1-type microglia marker iNOS and the proinflammatory factors TNF-α, IL-1β, and IL-6 at the mRNA level while promoting the mRNA expression of the M2-type microglia marker Arg1 and the anti-inflammatory factor, IL-10. These results indicate that CPT can promote the transformation of microglia/macrophages from the M1- to the M2-type and thus exerts neuroprotective effects. Researches have reported that CPT can inhibit neuroinflammation and improve cognitive dysfunction by acting on the NF-κB signaling pathway [56,57]. Therefore, we speculated that CPT may inhibit neuroinflammation and exert neuroprotective effects by inhibiting the NF-κB signaling pathway. In this study, we tested the levels of p-NF-κB and found that CPT inhibited the expression of p-NF-κB, suggesting that CPT may inhibit the polarization of M1 microglia and promote the polarization of M2 cells through the NF-κB signaling pathway.

In addition to the previous affective factors of CPT, there are some other functional molecular pathways that may be implicated in the antidepressant mechanism of CPT. For example, the monoamine oxidases (MAO), regulating the function of neurochemistry by degrading monoamine neurotransmitters (serotonin, dopamine, and norepinephrine), were deeply considered to be related to the psychiatric disorders, including depression [58]. In the meantime, the CPT was reported to bear prominent inhibitory potency against recombinant human MAO [59], which implies a potential molecular mechanism concerning the activity of CPT in depression by targeting the metabolism of monoamine neurotransmitters. Nevertheless, the detailed effect of CPT on the activity of MAO in mice and its role in the pathophysiology of depression need to be explored thoroughly in the future.

In summary, our study identified that CPT can improve depression-like behaviors in CUS-induced mice. The mechanisms may involve the restoration of hippocampal neurogenesis mediated by the BDNF/TrkB pathway and related signaling proteins, as well as the regulation of the polarization of microglia from M1 to M2 by the NF-κB signaling pathway, resulting in antineuroinflammatory activity. These results provide evidence for the underlying mechanism of the antidepressant-like effects of CPT and promote its consideration as a candidate drug for the treatment of depression and other related mental diseases.

## Abbreviations

Arg-1: arginase 1
BDNF: brain-derived neurotrophic factor
CPT: cryptotanshinone
CREB: cAMP-response element binding protein levels
CUS: chronic unpredictable stress
DCX: doublecortin
DG: dentate gyrus
ERK: extracellular regulated protein kinases
FST: forced swim test
FUST: female urine sniffing test
Iba-1: ionized calcium-binding adaptor molecule-1
IL: interleukin
iNOS: inducible nitric oxide synthase
MAO: monoamine oxidases
NF-κB: nuclear factor-κB
SPT: sucrose preference test
TrkB: tyrosine kinase receptor B
TNF-α: tumor necrosis factor α
